# The Part Task of the Part-Spacing Paradigm Is Not a Pure Measurement of Part-Based Information of Faces

**DOI:** 10.1371/journal.pone.0006239

**Published:** 2009-07-15

**Authors:** Qi Zhu, Xiaobai Li, Kari Chow, Jia Liu

**Affiliations:** 1 State Key Laboratory of Cognitive Neuroscience and Learning, Beijing Normal University, Beijing, China; 2 Graduate University of Chinese Academy of Sciences, Beijing, China; University of Leuven, Belgium

## Abstract

**Background:**

Faces are arguably one of the most important object categories encountered by human observers, yet they present one of the most difficult challenges to both the human and artificial visual systems. A variety of experimental paradigms have been developed to study how faces are represented and recognized, among which is the part-spacing paradigm. This paradigm is presumed to characterize the processing of both the featural and configural information of faces, and it has become increasingly popular for testing hypotheses on face specificity and in the diagnosis of face perception in cognitive disorders.

**Methodology/Principal Findings:**

In two experiments we questioned the validity of the part task of this paradigm by showing that, in this task, measuring pure information about face parts is confounded by the effect of face configuration on the perception of those parts. First, we eliminated or reduced contributions from face configuration by either rearranging face parts into a non-face configuration or by removing the low spatial frequencies of face images. We found that face parts were no longer sensitive to inversion, suggesting that the previously reported inversion effect observed in the part task was due in fact to the presence of face configuration. Second, self-reported prosopagnosic patients who were selectively impaired in the holistic processing of faces failed to detect part changes when face configurations were presented. When face configurations were scrambled, however, their performance was as good as that of normal controls.

**Conclusions/Significance:**

In sum, consistent evidence from testing both normal and prosopagnosic subjects suggests the part task of the part-spacing paradigm is not an appropriate task for either measuring how face parts alone are processed or for providing a valid contrast to the spacing task. Therefore, conclusions from previous studies using the part-spacing paradigm may need re-evaluation with proper paradigms.

## Introduction

There is a general consensus that the mechanisms involved in face processing are “special,” but there is less agreement as to what exactly constitutes this “specialness.” A newly developed paradigm, the part-spacing paradigm [1], has become increasingly popular in testing hypotheses for face specificity [2], [3] and in the diagnosis of face perception in cognitive disorders [4]–[9]. By either manipulating the shape of face parts (*i.e.,* the part task) or the fine distances among them (*i.e.,* the spacing task), the measurement afforded by this paradigm is presumed to provide information as to how the brain processes featural and configural information, respectively. The part task, along with the spacing task, provides a perfect tool for examining how featural information of faces is processed and interacted with configural information in the context of a whole face (*e.g.,* [3], [10]). However, we argue that when it is used to characterize how face parts alone are represented, the part task may confound pure information about face parts alone with the effect of face configuration on the perception of those parts.

Our daily experience in face recognition suggests the importance of using both featural and configural information to correctly identify a specific individual in a fraction of a second. The underlying mechanisms of processing these two types of information are hotly debated, however. A dominant view suggests that faces are encoded and processed as a gestalt, without an internal part structure (*i.e.,* Holistic-encoding hypothesis [11]–[13]). Recent fMRI and TMS studies, however, challenge this hypothesis by showing that featural information is encoded independent of the configural information, which supports a dual-mode hypothesis [14]–[19]. For example, a face-selective region in the lateral inferior occipital gyri (*i.e.,* occipital face area, OFA [20], [21]) is sensitive only to the presence of real face parts and not to the correct configuration of those parts [22], [see also 21]. Also, TMS stimulation of this region selectively disrupts subjects' ability to discriminate faces on the basis of differences in face parts but not on the basis of differences in the spacing among those parts [23].

**Figure 1:**
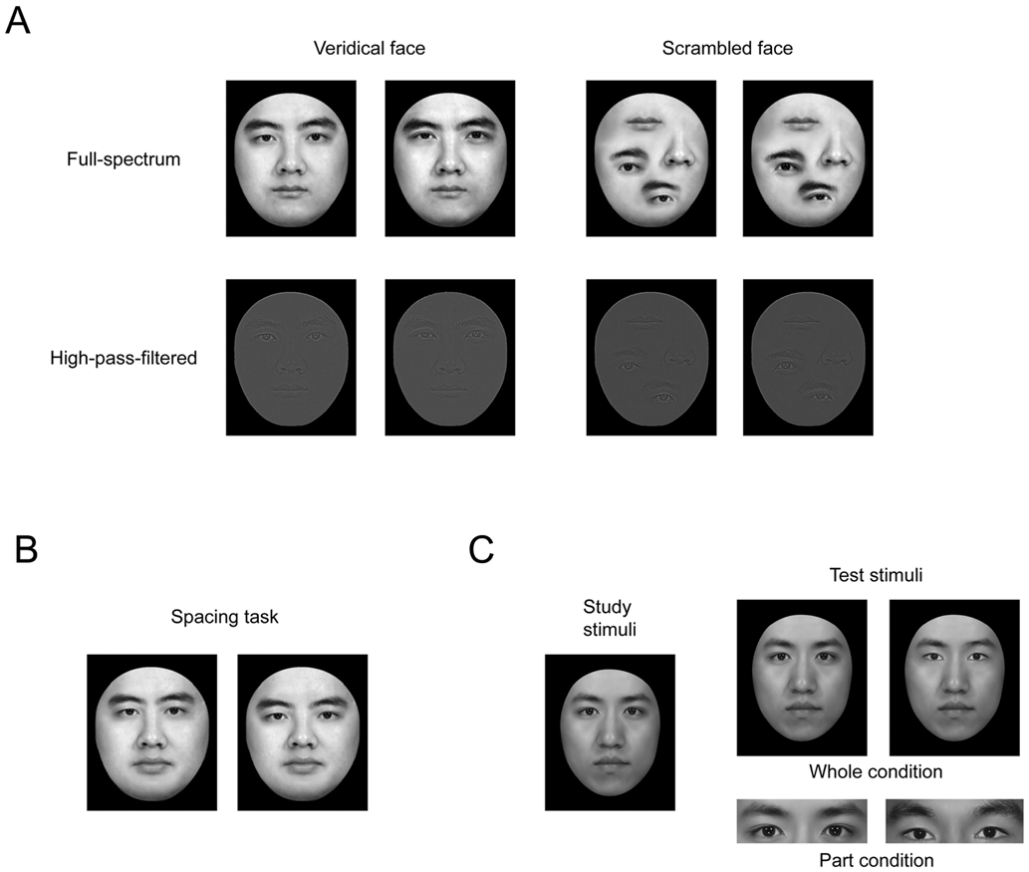
Stimulus. A) Face exemplars for four versions of part tasks that (i) either have veridical face configurations or do not, and (ii) either contain full-spectrum spatial frequencies, or contain only high spatial frequencies. Within each version of the part task, either the two eyes or the mouth are changed. B) Face stimuli for the spacing task. C) Face stimuli for whole-part task.

The dissociation in representing featural versus configural information in the brain suggests that the underlying mechanisms of processing the featural information should be studied outside the context of configural information. This is because evidence from the whole-part effect [12] and the composite effect [24] suggests that the discrimination of face parts is automatically influenced by an intact face configuration. Further, behavioral performance is a sum over outputs from all the stages involved, and therefore a measurement as regards face parts is likely to reflect effects from configural information, even if they had been processed at different stages and/or at different neural substrates. Therefore, to acquire pure information about face parts, the configural information must be removed. However, most recent studies on featural information processing have been carried out in the context of the veridical face configuration. In the part task of the part-spacing paradigm, the shape of face parts (either eyes or mouth) varies, but the first-order face configuration (*i.e.,* the “T”-shaped configuration of eyes above nose above mouth) and the second-order face configuration (*i.e.,* spacing) remain largely unchanged (Figure 1A, top left). This design may lead to conflicting results. For example, there is currently a debate as to whether the inversion of face stimuli affects the processing of the featural and configural information differently [25]. Some studies have shown that the size of the inversion effect on the featural information is as large as that on the configural information, using the part-spacing paradigm [2], [3], [26], [27], [ but see 1], [4], [19], [28]–[32]. Therefore, the featural information is proposed to be processed in a holistic fashion and not qualitatively different from that for configural information [3], [10]. We argue that the lack of qualitative difference in processing between featural and configural information may actually stem from problems in the design of the part task itself, as the inversion effect observed in the part task may actually reflect an additional contribution from face configuration. Specifically, in the part task of the part-spacing paradigm, the first-order face configuration is always present and, moreover, changing the shapes of face parts alters the second-order face configuration [16], [19], [33]. When face stimuli are inverted, the contribution from face configuration is eliminated, and therefore a decrease in accuracy is observed in behavioral performance.

To test this hypothesis, we either disturbed the processing of face configurations by rearranging face parts in a non-face configuration (Figure 1A, top right), or we kept the veridical face configuration but used high-pass-filtered face images, which is thought to reduce holistic face perception [34]–[36], [ but see 37]–[39] (Figure 1A, bottom left). The inversion effect on face parts in both scrambled faces and high-pass-filtered faces was compared to that for full faces. We predicted that the inversion effect should be either absent or significantly reduced when the holistic processing was interrupted. Finally, we further illuminated the role played by face configuration in the processing of face part information by testing a group of self-reported developmental prosopagnosic subjects [40]–[42] who are specifically impaired in holistic face perception.

## Methods

### Subjects

Nineteen subjects (ages 21–31; 11 males) with normal face perception and six prosopagnosic subjects (ages 19–20, 1 male) participated in the study. All subjects are right-handed and have normal or corrected-to-normal visual acuity. The prosopagnosic subjects were identified among college students at Beijing Normal University through a 21-item self-report face-recognition questionnaire followed by a one-hour semi-structured interview developed by Kennerknecht *et al.* [43]. None of the prosopagnosic subjects reported a history of severe head injury or neurological disease, but all complain of severe problems with face recognition in daily life. Because there are no well-accepted standards for screening developmental prosopagnosics, many studies are simply based on self-report and interview results [44]. However, we recognize that this approach is not ideal, and that objective behavioral tests may provide more conservative inclusion criteria to ensure the purity of prosopagnosics (*e.g.,* [3], [7], [45], [46]). The protocol was approved by the IRB of Beijing Normal University. Informed consent was obtained from all subjects before their participation.

### Stimulus and Procedure

#### General Procedure

Computer-based tasks were run on PC desktops using Matlab 6.5 with the psychophysics toolbox extensions [47], [48] at a viewing distance of approximately 70 cm from the screen. Two experiments were conducted for this study.

The first experiment included five component tests: one spacing task and four versions of part tasks. For the part tasks, subjects were instructed to discriminate part changes in face stimuli that (i) either had veridical face configurations, or did not, and (ii) contained either full-spectrum spatial frequencies, or only high spatial frequencies, and that were presented either upright or inverted. Before each of the component tests, subjects were explicitly informed as to what aspects of facial information were changed, and ten practice trials were given at the beginning of each block to ensure that the subjects understood the instruction and were familiar with the stimuli. Instructions were to respond as accurately as possible without sacrificing response speed.

The second experiment tested the prosopagnosic subjects with the whole-part task, the spacing task, and the part tasks with face configuration either preserved or scrambled. All subjects but one in the normal group participated in both experiments – one subject did not participate in the part tasks with high-pass-filtered faces.

#### Stimulus

The face stimuli used in these tests were gray-scale adult Chinese faces with external contour (a roughly oval shape with hair on the top and sides) removed. Three male faces were used to generate the stimuli for the part task, and all stimuli were 7 cm wide and 8.3 cm high (5.7°×6.8° visual degrees). Four sets of nine faces were generated from a face template containing eyebrows and nose. For the face set used in the standard part task of the part-spacing paradigm (Veridical), either the two eyes or the mouth were replaced in each of the nine faces by eyes and mouths of similar shape from three original male face images (Figure 1, top left). For the face set without veridical face configurations (Scrambled), the face parts from the nine faces in the veridical face set were rearranged in a non-face configuration. This non-face configuration was the same for all face stimuli in this set (Figure 1A, top right). For the high-spatial-frequency (HSF) face set, the above two face sets were Fourier-transformed and multiplied by high-pass Gaussian filters to preserve high spatial frequencies (above 40 cpf) (for details, see [35]).

Face stimuli for the spacing task were generated by varying the distance through either vertical displacement (between mouth and nose, 2 mm or 0.17°) or horizontal displacement (between two eyes, 3.4 mm or 0.28°) or both (horizontal displacement between two eyes, and vertical displacement between eyes and noses, 2.7 mm or 0.22°) (Figure 1B). The displaced face stimuli were likely in the normal range of anthropomorphic norms (vertical displacement between mouth and nose: 1.06 standard deviations (SD); between eyes and nose: 0.64 SD; horizontal displacement between two eyes: 2.21 SD) [49].

Another three male faces were used as targets in the whole-part task. Two distractor faces in whole face condition were created for each target face by either replacing the eyes, the mouth, or the nose from the target face with the corresponding feature from a different face. The target and distractor whole-face stimuli were 8.9 cm wide and 10.7 cm high (7.3°×8.7°). The individual face parts in the part condition were cropped from each of the target faces, creating a rectangular section with the feature in the center. The sizes of the face parts varied across different face parts, but the size for the same face part was constant (Figure 1C).

#### The Part Task

Two identical faces, or two faces that differed only in eyes or mouth, were presented sequentially, either upright or inverted. Subjects were instructed to judge whether the two faces were identical. Each trial started with a blank screen for 1 s, followed by the first face stimulus presented at the center of the screen for 500 ms. Then, after a blank interval of 1 s, the second stimulus was presented for 500 ms. Each response was followed immediately by a visual feedback that provided accuracy feedback. Eight conditions from a 2 (Veridical versus Scrambled)×2 (Full-spectrum versus HSF)×2 (Upright versus Inverted) design were tested in separate blocks (*i.e.,* 8 blocks in total). Tasks with stimuli with full-spectrum spatial frequencies were conducted before those with stimuli with only high spatial frequencies, and the test order of task with rest manipulation was counterbalanced across subjects. Each block included a total number of 72 trials, half of which consisted of identical faces.

#### The Spacing Task

The spacing task was also conducted in separate blocks so that the results could be compared directly with those of the part task. The procedure was the same as that of the part task, except that faces were either identical or they differed only in terms of the distances between parts.

#### The Whole-Part Task

This task had two phases. In the learning phase, subjects were instructed to memorize three faces and their associated names. Only when the subjects could correctly identify all face-name pairs were they allowed to enter the test phase. In the test phase, a question (*e.g.,* “Which is Xiao Zhang's nose (or mouth, or eyes)?”) was presented, followed by a choice of two alternative pictures presented to the left and right sides of the screen. The display was left on the screen until the subjects responded. There were two conditions, each consisting of 36 trials. For the part condition, the display contained two isolated features (*e.g.,* two noses): one was from the target face and the other from one of the learned faces. For the whole condition, the display contained two intact faces, with the target and the foil face differing only with respect to the individual feature that had been tested in the part condition; all other feature information was constant. These two conditions were randomly interleaved.

## Results and Discussion

**Figure 2:**
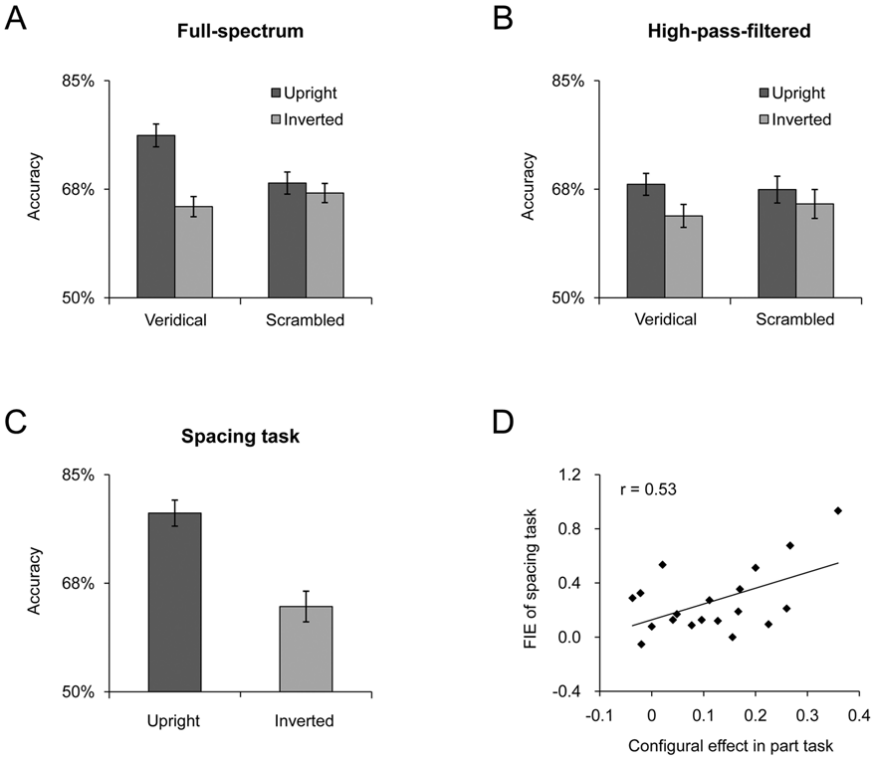
Behavioral results from normal subjects. A) Face inversion effect (FIE) for featural information when the first-order face configuration is either preserved (Veridical) or disturbed (Scrambled). Accuracy is shown on the y-axis and the error bar stands for standard error. B) Face inversion effect for featural information when face stimuli are high-pass-filtered. C) Face inversion effect for configural information in the spacing task. D) Correlation across subjects between the configural effect in the part task and the FIE of the spacing task.

We first examined whether there were qualitative differences in subjects' performance in discriminating part changes without the context of the veridical face configuration. The accuracy in discriminating part changes was analyzed in a two-way ANOVA for which the factors were stimulus type (Veridical versus Scrambled) and stimulus orientation (Upright versus Inverted). This ANOVA found significant main effects of stimulus type (F(1,18) = 6.09, p<.03) and stimulus orientation (F(1,18) = 25.37, p<.001). More importantly, a significant two-way interaction of stimulus type by stimulus orientation (F(1,18) = 15.10, p<.001) indicates that the amount of performance decrease in accuracy in discriminating face parts differs across stimulus type (Figure 2A). In fact, a *post-hoc* pair-wise *t*-test with Bonferroni correction showed that the inversion of face stimuli significantly decreased the accuracy of discriminating face parts when face configurations were intact (t(18) = 5.98, Bonferroni corrected p<.001) (Figure 2A, Veridical), replicating previous findings [2], [3], [26], [27], [30], [31]. Inverting the face parts in a non-face configuration, however, did not significantly decrease the accuracy of discriminating those face parts (t(18)<1) (Figure 2A, Scrambled). Further, the failure to observe an inversion effect was not due to a floor effect because the subjects' performance was significantly higher than that to be expected from chance (*i.e.,* 50%) whenever the faces were upright or inverted (*p*s<.001). Also, there was a significant drop in accuracy when the face configuration was scrambled than when it was intact, even when face stimuli were upright (t(18) = 4.92, Bonferroni corrected p<.001). Because the main difference between these two versions of the part task was the presence versus absence of the normal “T” face configuration, the difference in accuracy seems to have reflected an impact made by face configuration.

One may argue, though, that the absence of the inversion effect in the part task with scrambled faces may simply reflect a lack of experience with face stimuli that do not have veridical face configurations. To rule out this possibility, we examined subjects' behavioral performance in discriminating face parts when only high-spatial frequencies of face images were presented while face configurations were preserved (Figure 1A, bottom left). Previous studies have shown that face stimuli containing only high spatial frequencies are processed less holistically [35]. Therefore, we could expect that with HSF faces, the inversion effect for face parts in the context of face configurations would be significantly reduced and not significantly different from that when face configurations were scrambled. Indeed, a two-way ANOVA analysis revealed no significant interaction of stimulus type (Veridical versus Scrambled) by stimulus orientation (F(1,17) = 2.16, p = .16) when faces were high-pass-filtered (Figure 2B). The reduced inversion effect for face parts in the HSF faces was not due to a lack of experience, because low-spatial-frequency faces produced an even larger inversion effect than did full-spectrum faces [35]. Indeed, a significant three-way interaction of stimulus type (Veridical versus Scrambled), stimulus orientation (upright versus inverted), by spatial frequency (full-spectrum versus HSF) (F(1,17) = 7.62, p<.02) further indicates an additional contribution from face configuration on the perception of face parts in the part task. In other words, we found that by either rearranging face parts to a non-face configuration or by removing the low spatial frequencies of face images, the perception of face parts was no longer sensitive to face inversion. This suggests that there is a qualitative difference in processing configural and featural information (see also [25]).

Though our data show that the standard part task is not a pure measurement of part-based information, one might have argued that it could still serve as a contrast for the spacing task, so that researchers could investigate whether a manipulation (*e.g.,* inversion) produced a qualitative difference between configural changes and part changes. This argument is not tenable, either. When faces were inverted, a significant decrease in accuracy in discriminating configural changes was observed (t(18) = 5.65, p<.001) (Figure 2C); but this inversion effect was not significantly different from that for part changes in the standard part task (F(1,18) = 1.65, p = .22) (Figure 2A). On the other hand, the inversion effect for discriminating part changes in scrambled faces (Figure 2A) was significantly smaller than that for discriminating configural changes (F(1,18) = 12.87, p<.005). Therefore, the processing of featural information is indeed qualitatively different from the processing of configural information, but this difference is concealed by the presence of face configuration in the standard part task. In fact, the difference between these two versions of the part task, (Veridical−Scrambled)/Scrambled, was positively correlated with the inversion effect for configural changes in the spacing task, (Upright−Inverted)/Inverted, (r = 0.53, p<.02) (Figure 2D), suggesting again that the standard part task involves the processing of configural information rather than simply part-based analysis. Therefore, the standard part task cannot be used as a valid contrast to the spacing task, either.

**Figure 3:**
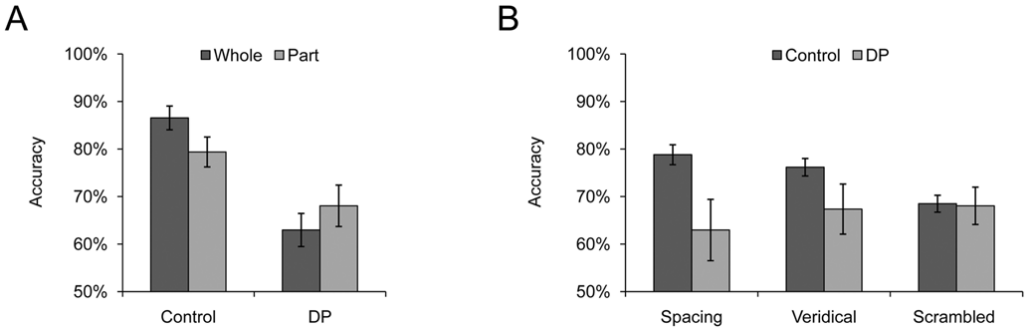
Behavioral results from prosopagnosic subjects. A) Whole-part effect from the normal (Control) and prosopagnosic (DP) groups. B) The application of the standard and modified part-tasks in measuring how face parts are processed in prosopagnosia (DP).

Because the part task of the part-spacing paradigm does not take into account the contribution of face configuration, the paradigm may be providing conflicting, or even false, information on face perception. For example, in a recent study in our lab on subjects with developmental prosopagnosia (DP) who show severe face perception deficits [50], the results from the whole-part task [12] and the part task of the part-spacing paradigm showed a conflicting pattern of deficits. The results from the whole-part task showed a significant two-way interaction of subject type (normal subjects versus DPs) by stimulus (whole versus part) (F(1,23) = 5.98, p<.03) (Figure 3A). This indicates that the normal controls were better at discriminating a specific face part in the context of a whole face than when the part was isolated (t(18) = 2.62, p<.02), whereas the DPs performance showed a part superiority effect (t(5) = 3.05, p<.03). This finding suggests that the DPs are selectively impaired in holistic processing but that their ability to identify isolated face parts is largely intact. The results from the part task of the part-spacing paradigm, however, could indicate that the DPs are impaired in their ability to process face parts, because the DPs' performance in identifying face parts was significantly poorer than that of the controls in this task (t(23) = 2.13, p<.05) (Figure 3B, Veridical). We suggest that these conflicting results are due to a failure to discount the contribution of face configuration in the part task. Indeed, when the DPs were instructed to discriminate face parts in a scrambled face, their performance was as good as that of the normal controls (t(23)<1) (Figure 3B, Scrambled). In fact, the DPs were impaired only in discriminating configural changes of faces, as the spacing task revealed (t(23) = 3.27, p<.005) (Figure 3B), and this is consistent with the findings from the whole-part task. Previous studies have revealed that individuals with developmental prosopagnosia may be selectively impaired in the holistic processing of faces [3], [6], [41], [46]. Consistent with these findings, we found that the DPs did not show the whole-part effect, and performed poorly in discriminating both configural and featural information in the context of a whole face. However, when face parts were presented either in isolation or outside the context of face configuration, the DPs' performance was as good as that of normal controls, suggesting that the processing of face parts is dissociable from the processing of face configurations.

In sum, converging evidence from both the inversion effect and the whole-part effect demonstrates that in the part task of the part-spacing paradigm confounds pure information about face parts alone with the face configuration on those parts. It is therefore not an appropriate task for either measuring how face parts alone are processed or for providing a valid contrast to the spacing task. That being said, we are not suggesting that conclusions from previous studies using the part-spacing paradigm are problematic, because the results from the part task do partially reflect an analysis of face parts as the task explicitly instructs. Rather, we simply suggest that, because of the possible influencing role of face configuration in the processing of face parts, conclusions drawn from studies using this paradigm may need further re-evaluation with proper paradigms.

## References

[1] Freire A, Lee K, Symons LA (2000). The face-inversion effect as a deficit in the encoding of configural information: Direct evidence.. Perception.

[2] Yovel G, Kanwisher N (2004). Face perception: Domain specific, not process specific.. Neuron.

[3] Yovel G, Duchaine B (2006). Specialized face perception mechanisms extract both part and spacing information: Evidence from developmental Prosopagnosia.. Journal of Cognitive Neuroscience.

[4] Le Grand R, Mondloch CJ, Maurer D, Brent HP (2001). Neuroperception - Early visual experience and face processing.. Nature.

[5] Le Grand R, Mondloch CJ, Maurer D, Brent HP (2003). Expert face processing requires visual input to the right hemisphere during infancy.. Nature Neuroscience.

[6] Duchaine BC (2006). Prosopagnosia as an impairment to face-specific mechanisms: Elimination of the alternative hypotheses in a developmental case.. Cognitive Neuropsychology.

[7] Le Grand R, Cooper PA, Mondloch CJ, Lewis TL, Sagiv N (2006). What aspects of face processing are impaired in developmental prosopagnosia?. Brain and Cognition.

[8] Nishimura M, Rutherford MD, Maurer D (2008). Converging evidence of configural processing of faces in high-functioning adults with autism spectrum disorders.. Visual Cognition.

[9] Shin YW, Na MH, Ha TH, Kang DH, Yoo SY (2008). Dysfunction in configural face processing in patients with schizophrenia.. Schizophrenia Bulletin.

[10] Yovel G, Kanwisher N (2008). The representations of spacing and part-based information are associated for upright faces but dissociated for objects: Evidence from individual differences.. Psychon Bull Rev.

[11] Farah MJ, Wilson KD, Drain M, Tanaka JN (1998). What is “special” about face perception?. Psychol Rev.

[12] Tanaka JW, Farah MJ (1993). Parts and wholes in face recognition.. Q J Exp Psychol A.

[13] Tanaka JW, Sengco JA (1997). Features and their configuration in face recognition.. Mem Cognit.

[14] Bartlett JC, Searcy J (1993). Inversion and configuration of faces.. Cogn Psychol.

[15] Searcy JH, Bartlett JC (1996). Inversion and processing of component and spatial-relational information in faces.. J Exp Psychol Hum Percept Perform.

[16] Rhodes G, Brake S, Atkinson AP (1993). What's lost in inverted faces?. Cognition.

[17] Macho S, Leder H (1998). Your eyes only? A test of interactive influence in the processing of facial features.. J Exp Psychol Hum Percept Perform.

[18] Cabeza R, Kato T (2000). Features are also important: Contributions of featural and configural processing to face recognition.. Psychological Science.

[19] Leder H, Bruce V (2000). When inverted faces are recognized: The role of configural information in face recognition.. Quarterly Journal of Experimental Psychology Section a-Human Experimental Psychology.

[20] Gauthier I, Tarr MJ, Moylan J, Skudlarski P, Gore JC (2000). The fusiform “face area” is part of a network that processes faces at the individual level.. Journal of Cognitive Neuroscience.

[21] Rossion B, Caldara R, Seghier M, Schuller AM, Lazeyras F (2003). A network of occipito-temporal face-sensitive areas besides the right middle fusiform gyrus is necessary for normal face processing.. Brain.

[22] Liu J, Harris A, Kanwisher N (2009). Perception of Face Parts and Face Configurations: An fMRI Study.. Journal of Cognitive Neuroscience.

[23] Pitcher D, Walsh V, Yovel G, Duchaine B (2007). TMS evidence for the involvement of the right occipital face area in early face processing.. Current Biology.

[24] Young AW, Hellawell D, Hay DC (1987). Configurational information in face perception.. Perception.

[25] Rossion B (2008). Picture-plane inversion leads to qualitative changes of face perception.. Acta Psychologica.

[26] Riesenhuber M, Jarudi I, Gilad S, Sinha P (2004). Face processing in humans is compatible with a simple shape-based model of vision.. Proceedings of the Royal Society of London Series B-Biological Sciences.

[27] Butler PD, Tambini A, Yovel G, Jalbrzikowski M, Ziwich R (2008). What's in a face? Effects of stimulus duration and inversion on face processing in schizophrenia.. Schizophrenia Research.

[28] Barton JJ, Keenan JP, Bass T (2001). Discrimination of spatial relations and features in faces: effects of inversion and viewing duration.. Br J Psychol.

[29] Mondloch CJ, Le Grand R, Maurer D (2002). Configural face processing develops more slowly than featural face processing.. Perception.

[30] Leder H, Carbon CC (2006). Face-specific configural processing of relational information.. British Journal of Psychology.

[31] Rhodes G, Hayward WG, Winkler C (2006). Expert face coding: Configural and component coding of own-race and other-race faces.. Psychonomic Bulletin & Review.

[32] Goffaux V, Rossion B (2007). Face inversion disproportionately impairs the perception of vertical but not horizontal relations between features.. J Exp Psychol Hum Percept Perform.

[33] Hosie JA, Ellis HD, Haig ND (1988). The effect of feature displacement on the perception of well-known faces.. Perception.

[34] Goffaux V, Hault B, Michel C, Vuong QC, Rossion B (2005). The respective role of low and high spatial frequencies in supporting configural and featural processing of faces.. Perception.

[35] Goffaux V, Rossion B (2006). Faces are “spatial”–holistic face perception is supported by low spatial frequencies.. J Exp Psychol Hum Percept Perform.

[36] Goffaux V (2008). The horizontal and vertical relations in upright faces are transmitted by different spatial frequency ranges.. Acta Psychologica.

[37] Boutet I, Collin C, Faubert J (2003). Configural face encoding and spatial frequency information.. Perception & Psychophysics.

[38] Collin CA, Liu CH, Troje NF, McMullen PA, Chaudhuri A (2004). Face recognition is affected by similarity in spatial frequency range to a greater degree than within-category object recognition.. Journal of Experimental Psychology-Human Perception and Performance.

[39] Gaspar C, Sekuler AB, Bennett PJ (2008). Spatial frequency tuning of upright and inverted face identification.. Vision Research.

[40] Kress T, Daum I (2003). Developmental prosopagnosia: A review.. Behavioural Neurology.

[41] Behrmann M, Avidan G (2005). Congenital prosopagnosia: face-blind from birth.. Trends in Cognitive Sciences.

[42] Duchaine BC, Nakayama K (2006). Developmental prosopagnosia: a window to content-specific face processing.. Current Opinion in Neurobiology.

[43] Kennerknecht I, Plumpe N, Edwards S, Raman R (2007). Hereditary prosopagnosia (HPA): the first report outside the Caucasian population.. Journal of Human Genetics.

[44] Gruter T, Gruter M, Carbon CC (2008). Neural and genetic foundations of face recognition and prosopagnosia.. J Neuropsychol.

[45] Duchaine B, Germine L, Nakayama K (2007). Family resemblance: Ten family members with prosopagnosia and within-class object agnosia.. Cognitive Neuropsychology.

[46] Duchaine B, Yovel G, Nakayama K (2007). No global processing deficit in the Navon task in 14 developmental prosopagnosics.. Social Cognitive and Affective Neuroscience.

[47] Brainard DH (1997). The Psychophysics Toolbox.. Spat Vis.

[48] Pelli DG (1997). The VideoToolbox software for visual psychophysics: transforming numbers into movies.. Spat Vis.

[49] Farkas LG (1981). Anthropometry of the Head and Face in Medicine.

[50] Li XB, Song YY (2007). First congenital prosopagnosia case reported in China.. Progress in Natural Science.

